# Perception of Stigma and Its Associated Factors Among Patients With Major Depressive Disorder: A Multicenter Survey From an Asian Population

**DOI:** 10.3389/fpsyt.2019.00321

**Published:** 2019-05-15

**Authors:** Yan Sun, Gang Chen, Li Wang, Nan Li, Manit Srisurapanont, Jin Pyo Hong, Ahmad Hatim, Chia-hui Chen, Pichet Udomratn, Jae Nam Bae, Yi-Ru Fang, Hong Choon Chua, Shen-Ing Liu, Tom George, Dianne Bautista, Edwin Chan, A. John Rush, Hong Yang, Yun-Ai Su, Tian-Mei Si

**Affiliations:** ^1^Institute of Mental Health, Peking University Sixth Hospital, Beijing, China; ^2^National Clinical Research Center for Mental Health Disorders and Key Laboratory of Mental Health, Ministry of Health, Peking University, Beijing, China; ^3^Department of Psychiatry, Shanxi Dayi Hospital, Taiyuan, China; ^4^Department of Psychiatry, Huai’an No.3 People’s Hospital, Jiangsu, China; ^5^Department of Epidemiology, Peking University Third Hospital, Beijing, China; ^6^Department of Psychiatry, Faculty of Medicine, Chiang Mai University, Muang, Thailand; ^7^Department of Psychiatry, University of Ulsan College of Medicine, Asian Medical Center, Seoul, South Korea; ^8^Department of Psychological Medicine, Faculty of Medicine, University of Malaya, Kuala Lumpur, Malaysia; ^9^Department of Psychiatry, Chang Gung Medical Center and Chang Gung University, Tao-Yuan, Taiwan; ^10^Department of Psychiatry, Faculty of Medicine, Prince of Songkla University, Songkhla, Thailand; ^11^Department of Psychiatry, Faculty of Medicine, Inha University Hospital, Incheon, South Korea; ^12^Division of Mood Disorders, Shanghai Mental Health Center, Shanghai Jiao Tong University School of Medicine, Shanghai, China; ^13^Institute of Mental Health, Woodbridge Hospital, Singapore, Singapore; ^14^Department of Psychiatry, Faculty of Medicine, Mackay Memorial Hospital, Taipei, Taiwan; ^15^North West Specialist Centre, Everton Park, QLD, Australia; ^16^Singapore Clinical Research Institute, Singapore, Singapore; ^17^Duke-National University of Singapore Graduate Medical School, Singapore, Singapore

**Keywords:** stigma, Asia, major depressive disorder, associated factors, social support

## Abstract

Stigma of major depressive disorder (MDD) is an important public health problem. This study aimed to examine the level of perceived stigma and its associated factors in MDD patients in five Asian countries, including China, Korea, Malaysia, Singapore, and Thailand. A total of 547 outpatients with MDD were included from Asian countries. We used the stigma scale of the Explanatory Model Interview Catalogue (EMIC) to assess stigma. The Montgomery–Asberg Depression Rating Scale (MADRS), Symptoms Checklist 90-Revised (SCL-90-R), Fatigue Severity Scale (FSS), Sheehan Disability Scale (SDS), 36-Item Short-Form Health Survey (SF-36), and Multidimensional Scale of Perceived Social Support (MSPSS) were used to assess symptoms, clinical features, functional impairment, health status, and social support. The stigma scores of patients under 55 years old were significantly higher than those equal to or greater than 55 years old (*P* < 0.001). The stigma scores exhibited significant negative correlation with age; MSPSS scores of family, friends, and others; and SF-36 subscale of mental health, but significant positive correlation with MADRS, FSS, SDS, and SCL-90-R subscale scores of depression, interpersonal sensitivity, obsession–compulsion, psychoticism, and somatization. Multivariate regression analysis revealed that age, SCL-90-R interpersonal sensitivity, obsession–compulsion, psychoticism, MSPSS scores of friends and others, and SF-36 of mental health were significantly associated with the level of perceived stigma. These findings suggest that MDD patients who are young, have a high degree of interpersonal sensitivity and psychoticism, have low health-related quality of life, and have low social support are the target population for stigma interventions in Asia.

## Introduction

Major depressive disorders (MDDs) are a very highly prevalent and seriously disabling public health problem worldwide. It has been estimated that, by 2020, MDD will become the second most common debilitating disease, trailing only cardiovascular disease (1). Data from the United States in 2002 indicated a 12-month prevalence of 6.6% for MDD and a lifetime prevalence of 16.2% (2). According to the latest China Mental Health Survey, the lifetime prevalence of MDD was 6.8% (3). However, less than 8% of individuals with MDD had ever sought any types of professional help in China (4). This treatment rate was significantly lower than that seen in Western countries, including America (5). High stigma has been considered as an important cause for the low rates of help seeking, lack of access to care, undertreatment, material poverty, and social marginalization (6). Stigma is regarded as a set of prejudices, stereotypes, discriminatory beliefs, and biases linked to the characteristics that differentiate a person from others (7). Mental health stigma can be conceptualized in a variety of ways, and it has usually been classified as perceived stigma and personal stigma. Perceived stigma concerns negative attitudes where one believes that society as a whole holds about individuals with mental illness, while personal stigma focuses on one’s own beliefs about individuals with mental illness (8). Stigma emerged as an important barrier to the treatment of depression and other mental illnesses. Discovering the associated factors of stigma may be a critical pathway for the development of public strategies and interventions to reduce stigma in an MDD population.

Previous studies have examined the risk factors of stigma in people with mental illnesses including MDD. Personal stigma associated with MDD was found to be greater among males and those with less education, lower prior contact with depression, and lower depression literacy (9). Psychological distress was associated with both personal stigma and perceived stigma (9). Up to now, these studies were mainly performed in MDD populations from European, American, and other Western countries (6, 9–11). Only a few studies in this field have been conducted in Asia. One study showed that stigma varied by age and coping styles (12). Another study found that cognitive behavioral therapy could effectively reduce stigma experienced by MDD patients (13). No studies have been conducted to systematically investigate factors associated with the MDD patients’ stigma.

Several questionnaires have been developed to assess stigma in healthy and clinical populations (14–19). In the 1990s, Weiss et al. (20) developed a framework for collecting data on five key themes of illness beliefs based on their work on leprosy in India, named the Explanatory Model Interview Catalogue (EMIC) (20). The EMIC probes multiple dimensions of illness belief. The stigma scale of EMIC was confirmed to have good validity and reliability for the investigation of perceived stigma in cross-cultural and cross-condition applicability, as evident from studies of onchocerciasis in Nigeria (21) and MDD (22), leprosy (23), mental health, and HIV (24) in India. Several studies have used the EMIC tool to examine illness beliefs in Chinese American immigrants. The results have shown that depressed Chinese American immigrants have a high stigma level, and nearly half have reported that they would conceal the nature of their problem from others (25). Genetic contamination, which jeopardizes the furthering of one’s family lineage, was found to be a culture-specific reason for strong stigma among Chinese American immigrants (26). Using EMIC, a recent study (18) examined the association between baseline stigma score and depression outcomes among Chinese American immigrants in a primary care setting. The study indicated that higher stigma scores at baseline have severe negative effects on depression outcomes, even after the patients have been diagnosed and agreed to treatment.

In this study, we used the stigma scale of EMIC (EMIC Stigma Scale) to examine perceived stigma and its associated factors in MDD patients from five Asian countries. We examined the correlates of stigma, including demographic variables, clinical features, social support, etc.

## Materials and Methods

### Subjects

This study used data from the Study on the Aspects of Asian Depression (SAAD) (27). This was a multicountry, cross-sectional, and observational study of MDD carried out in clinical settings in five Asian countries from 2008 to 2010. The inclusion criteria were as follows: 1) MDD diagnosis identified by using the Mini-International Neuropsychiatric Interview (MINI) (28), which meets the *Diagnostic and Statistical Manual of Mental Disorders* (*DSM-IV*) criteria; 2) males or females aged 18–65 years; and 3) the ability to read and understand the questionnaires and voluntarily sign written informed consent forms. The exclusion criteria included the following: 1) unstable medical condition, 2) a history of psychoactive substance abuse or dependence, 3) psychotic or bipolar disorder, 4) treatment with antipsychotic medication within the past 1 month, and 5) clinical diagnosis of dementia.

### Procedure

The study protocol was reviewed and approved by the independent ethics committees of the hospitals. All of the investigators completed consistency training on the use of the scales before the study began. After interrater agreement for the scales was confirmed to be satisfactory, enrollment of subjects was initiated in each country and site. All patients provided written informed consent before participating in the survey.

### Measures

Sociodemographic and clinical data were recorded using a self-report questionnaire, which included information about age, gender, education, occupation, marital status, and ethnicity. The following tools were used to assess stigma (EMIC stigma scale), clinical symptoms [Montgomery–Asberg Depression Rating Scale (MADRS), Symptoms Checklist 90-Revised (SCL-90-R), and Fatigue Severity Scale (FSS)], health status [36-Item Short-Form Health Survey (SF-36)], functional impairment [Sheehan Disability Scale (SDS)], and social support [Multidimensional Scale of Perceived Social Support (MSPSS)]. Except for the MINI and MADRS, all questionnaires were self-administered. Lundbeck Export A/S supervised the acquisition of versions in Chinese (both traditional and simplified), Korean, Malay, and Thai. A protocol for forward and backward translation was implemented to produce the equivalent translations of the FSS, MSPSS, and EMIC (27). All of these questionnaires are standardized and internationally validated instruments and have been widely used in previous studies (27, 29–32). The internal consistency of the instruments measured with Cronbach’s alpha ranged from 0.80 to 0.95 and was very good for all instruments.

**EMIC**: The EMIC is a semi-structured questionnaire that queries patients about multiple dimensions of illness behaviors and beliefs: chief complaint, conceptualization and labeling of illness, perceptions of stigma, causal attributions, and help-seeking patterns. The EMIC Stigma Scale is composed of 12 questions that were considered simple and useful for assessing perceived and experienced stigma. The details of each item are shown in **Table 1**. The scale with Likert scale response options is as follows: (3) “yes”; (2) “possibly”; (1) “uncertain”; (0) “no”. The answer “yes” indicates a strong and positive indication of stigma; therefore, it was assigned the highest value (3 points), while “no” indicates a strong and negative response, having been assigned the lowest value (0 point). Question 2 has a reverse score. The higher the sum of the scores, the greater the indication of stigma (20).

**Table 1:** Detailed items of the EMIC stigma scale.

1. If possible, would you prefer to keep people from knowing about this problem?
2. On the other hand, is there anyone in particular whom you would like to know about it?
3. Do you think less of yourself because of this problem?
4. Have you ever been made to feel ashamed or embarrassed (loss of face) because of your problem?
5. If they knew about it, would your neighbors, colleagues, or others in your community think less of you because of this problem?
6. Do you feel others have avoided you because of your problem?
7. Would some people refuse to visit your home because of this condition?
8. If they knew about it, would your neighbors, colleagues, or others in your community think less of the family because of this problem?
9. If others were to find out about your problem, might it cause problems for your family?
10. Would your family prefer to keep others from finding out about your condition?
11. (If you are unmarried) If people know about it, might this problem make it more difficult to marry? (If you are married) Might this condition cause problems in your marriage?
12. Could this problem make it more difficult for someone in your family to marry?

**MADRS** (33): The MADRS is a 10-item scale designed to assess the severity of core symptoms of MDD, i.e., apparent sadness, reported sadness, inner tension, reduced sleep, reduced appetite, concentration difficulties, lassitude, inability to feel, pessimistic thoughts, and suicidal thoughts. Each item is scored from 0 to 6. A score of 0 indicates the absence of symptoms, and a score of 6 indicates the most severe symptoms. All items are summed to provide a total score, with higher total scores indicating more severe MDD.

**SCL-90-R** (34): The SCL-90-R is a 90-item inventory and contains a wide range of symptomatological content on mental illness, such as feelings, emotions, thinking, behavior, habits, interpersonal relationships, appetite, and sleep. These items are further divided into nine subscales, i.e., somatization, obsession–compulsion, interpersonal sensitivity, depression, anxiety, hostility, phobic anxiety, paranoid ideation, and psychoticism. Each of the items is scored from 1 to 5 (1 = no, 2 = mildly, 3 = moderately, 4 = severe, 5 = extremely).

**FSS** (35): FSS is a nine-item questionnaire that assesses the severity of fatigue related to physical functioning, exercise, work, family, and social life. Each item is scored from 1 (representing no fatigue) to 7 (representing extreme fatigue). All items were averaged to indicate the severity of fatigue.

**SDS** (36): The SDS is a three-item scale designed to assess disability in three domains of the patient’s life: work and/or school, social life/leisure activities, and family life/home responsibilities. Each item is rated from 0 (indicating not at all) to 10 (indicating extreme disability), with scores of 1–3, 4–6, 7–9, and 10 indicating mild, moderate, marked, and extreme disability, respectively. All items were summed to provide a total score ranging from 3 to 30, which was used to reflect the disability level.

**SF-36** (37): The SF-36 is a questionnaire that measures self-perceived general health and quality of life across eight health domains, including physical functioning, role limitations due to physical health, bodily pain, general health perceptions, vitality, social functioning, role limitations due to emotional problems, and mental health. Higher scores in each subscale indicate better health. This study used the acute version, which required particular situations within the previous 1-week period to be recalled.

**MSPSS** (38): The MSPSS is a brief social support tool designed to measure the respondent’s perception of the adequacy of support he/she receives from three kinds of sources: a significant other, family, and friends. It is a 12-item scale, and each scale is scored from 0 to 6. Higher scores indicate greater perceived social support.

### Statistical Analysis

All analyses were performed using SPSS software package (version 19.0; SPSS Inc, Chicago, IL, USA). The distribution of the data was examined with the Kolmogorov–Smirnov test. The demographic and clinical data were summarized as mean (or ± SD) for continuous variables with normal distributions. Medians (or min–max ranges) and nonparametric tests were used for continuous variables with non-normal distributions and categorical variables. Pairwise comparison was used to analyze the EMIC stigma scores with respect to gender, age, education level, marital status, and employment status. We used Pearson’s correlation analysis to examine the correlation between stigma scores and sociodemographic and clinical variables. Additionally, multiple regression analysis was employed to investigate the extent to which each of the correlated factors (*P* < 0.1) predicted the level of stigma.

## Results

Of 1,917 outpatients who were screened for eligibility, 637 (33.2%) were eligible. Of the 637 outpatients, 556 were enrolled in the study. The remaining 81 outpatients were not enrolled due to refusal or unwillingness to cooperate (*n* = 58) or insufficient patience to be interviewed (*n* = 14) or insufficient time to participate (*n* = 9). After interviews, nine participants were excluded from further analysis because the site investigator judged them as not having MDD. A total of 547 patients with MDD were enrolled from mainland China (114 cases), Taiwan (99 cases), Singapore (40 cases), Korea (101 cases), Thailand (103 cases), and Malaysia (90 cases). The demographic and clinical data are provided in **Table 2**.

**Table 2:** Comparisons of the stigma level of MDD patients with different sociodemographic and clinical features.

	*n* (%)	EMIC			
		Mean	SD	T/F	P
**Gender**	547			0.444	0.657
Male	195 (35.6)	14.49	9.16		
Female	352 (64.4)	14.14	8.59		
**Age (years)**	547			5.049	<0.001***
<55	448 (81.9)	15.14	8.68		
≥55	99 (18.1)	10.2	8.21		
**Ethnicity**	547			3.252	0.002*
Caucasian	0 (0)				
Chinese-cn	114 (20.8)	16.72	9.39		
Chinese-tw	99 (18.1)	12.22	8.13		
Chinese-my/sg	77 (14.1)	15.38	8.78		
Korea	101 (18.5)	14.13	8.67		
Thai	102 (18.6)	12.03	7.74		
Indian	24 (4.4)	15.17	9.98		
Malay	27 (4.9)	15.22	9.03		
Others	3 (0.5)	17.00	7.00		
**Education**	547			−3.979	<0.001***
≤Primary school	80 (14.6)	10.65	7.78		
≥Middle school	467 (85.4)	14.88	8.82		
**Marital status**	546			3.318	0.011*
Unmarried	160 (29.3)	15.70	8.66		
Married	285 (52.2)	13.65	8.83		
Cohabited	33 (6.0)	11.97	7.93		
Widowed	23 (4.2)	10.63	7.59		
Separated/divorced	45 (8.2)	16.20	8.86		
**Employment status**	541			2.138	0.038*
Full time	233 (43.1)	15.12	9.11		
Part time	24 (4.4)	12.87	7.60		
Homemaker	113 (20.9)	13.78	7.84		
Student	70 (12.9)	14.03	7.95		
Retired	45 (8.3)	10.76	8.25		
Sick leave ˃3 months	28 (5.2)	17.44	11.25		
Disability	9 (1.7)	10.67	8.96		
Unemployed	19 (3.5)	14.06	10.46		
**M.I.N.I. MDD subtype**				0.697	0.485
MDD with melancholia	370 (67.6)	13.88	8.42		
MDD with non-melancholia	177 (32.4)	14.45	8.97		

MDD, major depressive disorder; MINI, Mini-International Neuropsychiatric Interview; CN, China; MY/SG, Malaysia/Singapore; TW, Taiwan.

*P < 0.05; ***P < 0.001.

As shown in **Table 2**, stigma scores for MDD patients showed significant differences with respect to different age group (*t* = 5.049, *P* < 0.001), marital status (*F* = 3.318, *P* = 0.011), occupation (*F* = 2.138, *P* = 0.038), educational level (*F* = −3.979, *P* < 0.001), and ethnicity (*F* = 3.252, *P* = 0.002). We did not find significant differences between MDD with melancholia and MDD with non-melancholia subtype (*t* = 0.697, *P* = 0.485).

The relationship between clinical variables and the EMIC stigma scores is shown in **Table 3**. Stigma scores showed significant negative correlations with the following variables: age (*r* = −0.193, *P* < 0.001); MSPSS subscales family (*r* = −0.224, *P* < 0.001), friends (*r* = −0.192, *P* < 0.001), and significant others (*r* = −0.132, *P* = 0.002); and mental health (*r* = −0.266, *P* < 0.001) and social function (*r* = −0.237, *P* < 0.001) subscales of the SF-36. Stigma scores showed significant positive correlations with MADRS (*r* = 0.254, *P* < 0.001); FSS (*r* = 0.147, *P* = 0.001); SDS (*r* = 0.251, *P* < 0.001); and SCL-90-R subscales of depression (*r* = 0.341, *P* < 0.001), interpersonal sensitivity (*r* = 0.340, *P* < 0.001), obsession–compulsion (*r* = 0.220, *P* < 0.001), psychoticism (*r* = 0.320, *P* < 0.001), and somatization (*r* = 0.099, *P* = 0.023).

**Table 3:** Pearson correlation analysis of stigma-related factors.

	Median (G1, G3)	EMIC	
		*r*	*P*
Age	36 (25, 48)	−0.193	<0.001***
FSS-total	5.3 (4.3, 6.1)	0.147	0.001**
MADRS-total	29 (24, 35)	0.254	<0.001***
MSPSS-family	5.0 (3.5, 6.0)	−0.224	<0.001***
MSPSS-friends	4.4 (3.1, 5.4)	−0.192	<0.001***
MSPSS-significant others	5.0 (3.3, 6.0)	−0.132	0.002**
SCL-90-R-depression	2 (1, 3)	0.341	<0.001***
SCL-90-R-interpersonal sensitivity	1.3 (0.6, 2.1)	0.340	<0.001***
SCL-90-R-obsessive compulsion	1.9 (1.2, 2.5)	0.220	<0.001***
SCL-90-R-psychoticism	1.0 (0.5, 1.6)	0.320	<0.001***
SCL-90-R-somatization	1 (1, 2)	0.099	0.023*
SDS-total	18 (12, 24)	0.251	<0.001***
SF-36-mental health	30 (15, 45)	−0.266	<0.001***
SF-36-social function	50 (25, 63)	−0.237	<0.001***

*P < 0.05; **P < 0.01; ***P < 0.001.

The results of the multiple regression analysis are shown in **Table 4**. The following variables were significant contributors to MDD stigma scores: age (*b* = 2.094, *P* = 0.037); the MSPSS subscales of friends (*b* = −1.421, *P* = 0.001) and significant others (*b* = 1.062, *P* = 0.003); the SCL-90-R subscales of interpersonal sensitivity (*b* = 1.572, *P* = 0.014), obsession–compulsion (*b* = −1.630, *P* = 0.018), and psychoticism (*b* = 1.610, *P* = 0.032); and the mental health (*b* = −0.063, *P* = 0.011) subscale of the SF-36.

**Table 4:** Multivariate regression analysis of stigma-related factors.

Independent variables	EMIC			
	Regression coefficients	95% confidence interval	*t*	*P*
Age (<55)	2.094	0.131, 4.056	2.096	0.037*
Age (≥55)	0^a^			
Education (≤primary school)	−2.024	−4.108, 0.060	−1.908	0.057
Education (≥middle school)	0^a^			
Unmarried	−1.847	−4.566, 0.872	−1.334	0.183
Married	−1.424	−4.047, 1.199	−1.066	0.287
Cohabited	−2.317	−6.175, 1.541	−1.180	0.239
Widowed	−4.202	−8.521, 0.117	−1.911	0.057
Separated/divorced	0^a^			
MADRS-total	0.603	0.044, 0.170	1.155	0.249
MSPSS-family	−0.420	−0.889, 0.048	−1.762	0.079
MSPSS-friends	−1.421	−2.22, −0.622	−3.493	0.001**
MSPSS-significant others	1.062	0.372, 1.751	3.026	0.003**
SCL-90-R-depression	0.989	−0.661, 2.639	1.177	0.240
SCL-90-R-interpersonal sensitivity	1.572	0.320, 2.824	2.467	0.014*
SCL-90-R-obsessive compulsion	−1.630	−2.974, −0.286	−2.383	0.018*
SCL-90-R-psychoticism	1.610	0.136, 3.085	2.146	0.032*
SDS-total	0.042	−0.065, 0.148	0.765	0.444
SF-36-mental health	−0.063	−0.111, −0.015	−2.567	0.011*

R^2^ = 0.297 (adjust R^2^ = 0.266). MADRS, Montgomery–Asberg Depression Rating Scale; MSPSS, Multidimensional Scale of Perceived Social Support; SCL-90-R, Symptoms Checklist 90-Revised; SDS, Sheehan Disability Scale; SF-36, 36-Item Short-Form Health Survey.

a This parameter is redundant and is set to zero.

*P < 0.05, **P < 0.01.

## Discussion

This is a multicountry, multicenter study on stigma and associated factors among Asians with MDD. Among 547 MDD patients from five Asian countries, we found significant negative correlations between the EMIC stigma scores and age, mental health, quality of life as assessed by the SF-36, and several domains of social support as assessed by MSPSS. Significant positive correlations were found between the EMIC stigma scores and depressive severity (total scores on the MADRS and the depression subscale of the SCL-90-R), certain psychological characteristics (interpersonal sensitivity, obsessive–compulsion, psychoticism, and somatization as assessed by SCL-90-R), fatigue degree, and social functioning as assessed by the SDS. Among these, multiple regression analysis revealed that age, clinical symptoms (including interpersonal sensitivity, obsession–compulsion, and psychoticism), health-related quality of life across mental health status, and social support from friends and important others were significantly associated with patients’ stigma.

We found that stigma level of individuals younger than 55 was significantly higher than that of patients 55 or older. This effect of age on MDD patients’ stigma was consistent with a previous study that showed greater stigma among younger people (10). The multiple regression analysis conducted in our study also showed that being younger than 55 was an important risk factor for the formation of stigma. One possible explanation is that people younger than 55 usually have more pressure or responsibility stemming from many social and life facets, such as employment, education, and family, which make them more prone to conceal their illness for fear of losing their job and failing to integrate into society.

We also found significant correlations between the EMIC stigma scores and the SCL-90-R subscales of depression, interpersonal sensitivity, obsession–compulsion, somatization, and psychoticism. Further analysis showed that the interpersonal sensitivity, obsession–compulsion, and psychoticism subscales were significantly associated with MDD stigma. These results suggest that interpersonal sensitivity and psychoticism are risk factors for MDD stigma but that obsession–compulsion is a protective factor against stigma. Consistent with our study, many studies have shown that people with higher interpersonal sensitivity and psychoticism had higher levels of stigma (39, 40). The significant positive correlation between the EMIC stigma level and obsession–compulsion scores was not consistent with previous studies (2, 41), which have shown that patients’ desire to not disclose their symptoms to other people may be due to fear and having encountered stigmatization in different areas of their lives. Possible reasons for this inconsistency may include the following. 1) The obsession–compulsion symptoms are always spontaneously fluctuating over time. Additionally, one study reported that 55.2% of patients with obsessive–compulsive disorder (OCD) believed that they could overcome symptoms by themselves (42). These two factors easily contribute to patients believing that obsession–compulsion symptoms are not associated with a true illness. 2) Delaying treatment because of perceived social stigma was only endorsed by 12.5% of patients. Another study showed that a failure to control thoughts and behaviors was endorsed by a large percentage of patients with OCD as a motivator for seeking help (43). 3) This study revealed that the fact that most patients with OCD are willing to actively search for help may support adequate awareness of their symptoms and thus ultimately a solution to their problems (43). Further, we found that the mental health subscale of the SF-36 was significantly associated with stigma. The quality of life in MDD patients was diminished (35). People who worry about stigmatization are also more prone to withdraw from social contacts and choose a more isolated existence to avoid the risk of rejection or discrimination, which may in turn lead to further demoralization, low income, unemployment, and restricted social networks. This study found that social support was significantly correlated with MDD patients’ stigma. Multivariate regression analysis showed that the social support subscale of “significant others” was significantly associated with stigma formation, but it was a positive correlation. Possible reasons for this are that participants with higher stigma may be more unwilling to seek the support of family and friends, but they may actively or passively seek help from “significant others,” such as a physician. The results of this study provided further support for the importance of reducing stigma and discrimination against people with MDD.

There were several limitations of the current study. First, the cross-sectional design limits the inference of causality of stigma and its associated factors, investigation of which may be feasible with a longitudinal follow-up study. Second, participants in this study mainly came from urban samples in Asian countries, and nearly half of the participants came from the mainland of China. Thus, the present sample does not represent the overall MDD population, and it unavoidably biases the results. Third, although the multiple regression analysis showed that only some factors contributed to stigma, many other factors might affect MDD-related stigma, such as personality traits, treatment types, and religious beliefs. Future studies should examine the associated factors of MDD-related stigma in a broader range of variables.

In summary, this study provides evidence on stigma level and its associated factors in a large sample of Asian patients with MDD. These findings suggest that patients who are young, have a high degree of interpersonal sensitivity and psychoticism, have low health-related quality of life, and have low social support have greater perceptions of MDD-related stigma, suggesting that these patients are the target population for stigma interventions. Finally, our study provides empirical cues for the development of public intervention strategies to reduce stigma in MDD population in Asia.

## Ethics Statement

The study protocol was reviewed and approved by the independent ethics committees of the hospitals. All patients provided written informed consent before participating in the survey.

## Author Contributions

The data analysis was done by YS, GC, LW, NL, Y-AS, and T-MS. The manuscript was written by YS, GC, LW, HY, Y-AS, and T-MS. All the authors reviewed and revised the manuscript and approved to submit it to this journal.

## Funding

This study was supported by unrestricted research grants from Lundbeck A/S and the Duke-National University of Singapore Office of Clinical Research. Lundbeck A/S had no role for research conception, study design, data analysis, or study report. Duke-National University of Singapore Office of Clinical Research had the role for the quality control of the study and data preliminary analysis. This research was also supported by the National Natural Science Foundation of China (No. 81630031), Capital Medical Development Research Fund (2016-1-4111), Beijing Municipal Science and Technology Project (Z171100000117016), and National Key Technology R&D Program (2015BAI13B01).

## Conflict of Interest Statement

The authors declare that the research was conducted in the absence of any commercial or financial relationships that could be construed as a potential conflict of interest.
